# FireProt^DB^ 2.0: large-scale manually curated database of the protein stability data

**DOI:** 10.1093/nar/gkaf1211

**Published:** 2025-11-20

**Authors:** Milos Musil, Simeon Borko, Joan Planas-Iglesias, David Lacko, Monika Rosinska, Petr Kabourek, Lígia O Martins, Mateusz Tataruch, Jiri Damborsky, Stanislav Mazurenko, David Bednar

**Affiliations:** Loschmidt Laboratories, Department of Experimental Biology and RECETOX, Masaryk University, 625 00, Brno, Czech Republic; Department of Information Systems, Faculty of Information Technology, Brno University of Technology, 612 66, Brno, Czech Republic; International Clinical Research Centre, St. Anne’s University Hospital Brno, 602 00, Brno, Czech Republic; Loschmidt Laboratories, Department of Experimental Biology and RECETOX, Masaryk University, 625 00, Brno, Czech Republic; International Clinical Research Centre, St. Anne’s University Hospital Brno, 602 00, Brno, Czech Republic; Loschmidt Laboratories, Department of Experimental Biology and RECETOX, Masaryk University, 625 00, Brno, Czech Republic; International Clinical Research Centre, St. Anne’s University Hospital Brno, 602 00, Brno, Czech Republic; Loschmidt Laboratories, Department of Experimental Biology and RECETOX, Masaryk University, 625 00, Brno, Czech Republic; International Clinical Research Centre, St. Anne’s University Hospital Brno, 602 00, Brno, Czech Republic; International Clinical Research Centre, St. Anne’s University Hospital Brno, 602 00, Brno, Czech Republic; Loschmidt Laboratories, Department of Experimental Biology and RECETOX, Masaryk University, 625 00, Brno, Czech Republic; International Clinical Research Centre, St. Anne’s University Hospital Brno, 602 00, Brno, Czech Republic; Instituto de Tecnologia Química e Biológica António Xavier, Universidade Nova de Lisboa, 2780-157, Oeiras, Portugal; Jerzy Haber Institute of Catalysis and Surface Chemistry, Polish Academy of Sciences, PL-30239, Krakow, Poland; Loschmidt Laboratories, Department of Experimental Biology and RECETOX, Masaryk University, 625 00, Brno, Czech Republic; International Clinical Research Centre, St. Anne’s University Hospital Brno, 602 00, Brno, Czech Republic; Loschmidt Laboratories, Department of Experimental Biology and RECETOX, Masaryk University, 625 00, Brno, Czech Republic; International Clinical Research Centre, St. Anne’s University Hospital Brno, 602 00, Brno, Czech Republic; Loschmidt Laboratories, Department of Experimental Biology and RECETOX, Masaryk University, 625 00, Brno, Czech Republic; International Clinical Research Centre, St. Anne’s University Hospital Brno, 602 00, Brno, Czech Republic

## Abstract

Thermostable proteins are crucial in numerous biomedical and biotechnological applications. However, naturally occurring proteins have evolved to function in mild conditions, and laboratory experiments aiming at improving protein stability have proven laborious and expensive. Computational methods overcome this issue by providing a cheap and scalable alternative. Despite significant progress, their reliability is still hindered by the availability of high-quality data. FireProtDB 2.0 (http://loschmidt.chemi.muni.cz/fireprotdb) is a large-scale database aggregating stability data from multiple sources. The second version builds upon its predecessor, retaining its original functionality while introducing a new approach to data storage and maintenance. The new scheme enables the introduction of both absolute and relative data types connected with measurements of wild-types, mutants, protein domains, and *de novo* designed proteins. Furthermore, while the original database was limited to single-point mutations, more complex data such as insertions, deletions, and multiple-point mutations are now available. As a result, the inclusion of large-scale mutagenesis has increased the size of the database from 16 000 to almost 5 500 000 experiments. Moreover, the updated abstract scheme is fully expandable with any new measurements and annotations without the need for any restructuring. Finally, the tracking of history together with fixed identifiers is in accordance with the FAIR principles.

## Graphical Abstract

**Graphical Abstract:**
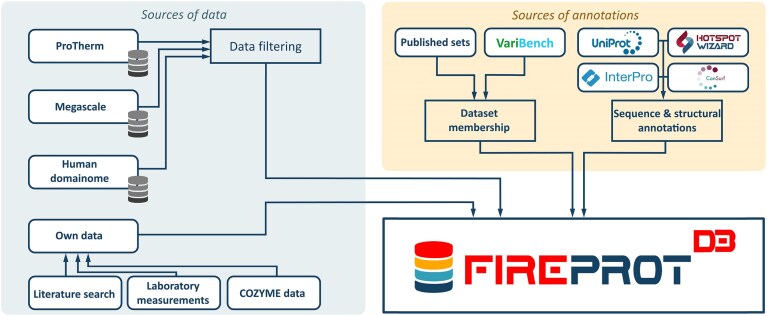

## Introduction

Proteins play essential roles in a wide range of biomedical, pharmaceutical, and biotechnological applications. However, most naturally occurring proteins have evolved to function under relatively mild physicochemical conditions [1] and are therefore not inherently suited for the harsh environments typical of industrial processes. Introducing mutations that enhance protein stability enables their use under non-natural conditions, such as elevated temperatures [2] or extreme pH levels [3]. Increased protein stability is also associated with improved serum survival time [4], higher expression yields [5], and prolonged activity in the presence of denaturing agents [6]. Traditionally, the effects of mutations on protein stability are assessed through laboratory experiments, which, while reliable, are time-consuming, costly, and limited in scalability. As a result, only a small portion of the mutational space can be experimentally explored.

Over the past two decades, numerous predictive tools have been developed to reduce the vast mutational space to a more manageable subset [7]. These tools use rapid and moderately accurate computational methods to prioritize potentially stabilizing mutations for experimental validation. In general, computational approaches can be classified into three main categories: (i) force-field methods estimating protein free energy by calculating or approximating individual terms in an energy equation [8, 9]; (ii) evolution-based methods deriving insights from multiple sequence alignments and phylogenetic analyses [10, 11]; and (iii) machine learning methods extracting informative features from available data to make predictions [12−14].

While force-field methods are generally considered more robust, their advancement is limited by the current understanding of molecular interactions and the high computational cost involved. In contrast, machine learning offers a faster, more scalable alternative capable of uncovering novel, previously unknown patterns in data [15]. As a result, machine learning techniques have advanced rapidly—from simple neural networks [16, 17] to convolutional architectures [18, 19], and most recently to large-scale transformer-based language models [20−22]—all within a few decades. However, these models require large volumes of high-quality data, which can be challenging to obtain, particularly in the areas with complex biological data, such as protein stability [23, 24]. Furthermore, independent validation sets are essential for the rigorous evaluation of newly developed predictive tools [25].

To address this issue, the first version of FireProtDB [26] was released in 2021 as a suggested source of data for the training of novel predictive tools. FireProtDB addressed the previously reported errors and inaccuracies [15, 27] present in the older datasets and databases by thorough manual curation. It also enriched the data with several sequence- and structure-based annotations, all readily prepared to be utilized by researchers to develop and benchmark their tools. Despite the wide use by the community, the original FireProtDB database had a number of limitations. It was designed around single-point mutations, fixed structures, and less-scalable technologies, which did not allow for an easy expansion to new types of data and for effective searching in large datasets. It also could not store mutations in *de novo* designed protein sequences, which are becoming more abundant due to the recent progress in AI-based protein design [28, 29].

The herein presented second version of the database builds upon its predecessor by retaining its original functionality while introducing a more abstract and flexible data structure. This new structure supports the storage of entirely new data types, annotations, and measurements, and is designed to be easily extendable for future updates. In addition, through the integration of multiple data sources [25, 30, 31] and large-scale mutagenesis studies [32], the size of the FireProtDB database has increased by two orders of magnitude—from fewer than 16 000 to over 5 million experiments, encompassing almost 13 million measurements. Combined with a fully redesigned interactive user interface enabling intuitive visualization of diverse data types and an optimized search engine capable of constructing complex queries, FireProtDB now serves as a powerful resource for various tasks. It is well-suited for both researchers aiming to build independent datasets for training, validation, and benchmarking their machine learning models, and for those simply seeking available data on their protein of interest.

## Materials and methods

### Data sources

The first version of the FireProtDB database provided an accessible solution for storing protein stability data and constructing training and validation datasets for machine learning applications. However, it was limited to single-point substitutions and supported only a small, predefined set of annotations and experimental measurements. The updated version supports both single- and multiple-point substitutions, as well as insertions, deletions, and measurements related to wild-type sequences. It can effectively store both absolute values (associated with the protein, such as ΔG and T_m_) and relative values (associated with the mutation, such as ΔΔG and ΔT_m_). Furthermore, the database is fully extensible as all measurements and annotations are stored as abstract key-value entities, allowing seamless integration of new data types as they become available.

In total, the second version of the FireProtDB database comprises 12 923 886 measurements, aggregated into 5 465 664 experiments across 2 762 unique proteins. It is important to note that duplicate measurement values may occur; however, these represent results obtained under different experimental conditions—such as variations in pH, method, or buffer concentration—or from independent data sources. Such cases are automatically treated as separate experiments. Currently, ∼6% of mutations are present in more than one dataset, and only about 40 data points have multiple measurements of the same type but recorded under distinct conditions. The data originate from four main sources:

- The ProTherm database [31] was the original source of much of the experimental data stored in the older version of FireProtDB. It includes both single- and multiple-point substitutions, as well as measurements for wild-type proteins. In addition to protein stability data, ProTherm contained a variety of other experimental measurements—such as ΔCp, ΔH, and *m*-values—along with detailed descriptions of experimental conditions, including pH, denaturant concentration, and measurement methods. After filtering out problematic entries (e.g. mutations with incorrect residue mapping or inconsistent data formatting), 6 691 single-point and 1 470 multiple-point substitutions were retained, along with 12 049 entries describing wild-type protein measurements.
- The MegaScale dataset [30] comes from one of the largest experimental studies of protein folding stability, generated using high-throughput complementary DNA-display proteolysis. Although the dataset is limited to small protein domains and relies on protease-derived stability proxies rather than direct thermodynamic measurements, it remains a valuable source of large-scale mutational data. After applying quality filters, the dataset contributed 22 901 deletions, 47 173 insertions, 486 666 single-point, and 184 642 double-point substitutions. It also enriched the database with 148 *de novo* designed proteins.
- The Human Domainome 1 dataset [32] originates from a large-scale site-saturation mutagenesis study involving over 500 human protein domains, evaluated using cellular abundance assays. Two of its subsets (Supplementary Datasets 2 and 5) were incorporated into FireProtDB due to their relevance to protein stability. This added 4 286 592 single-point substitutions, and 27 545 deletions to the database.
- The remaining data were gathered from various sources, including internal datasets generated by the Loschmidt Laboratories [33] and comprehensive literature mining. Moreover, 165 additional data points [34–42] were obtained through collaboration with the members of the COZYME network [43].

**Figure 1.**
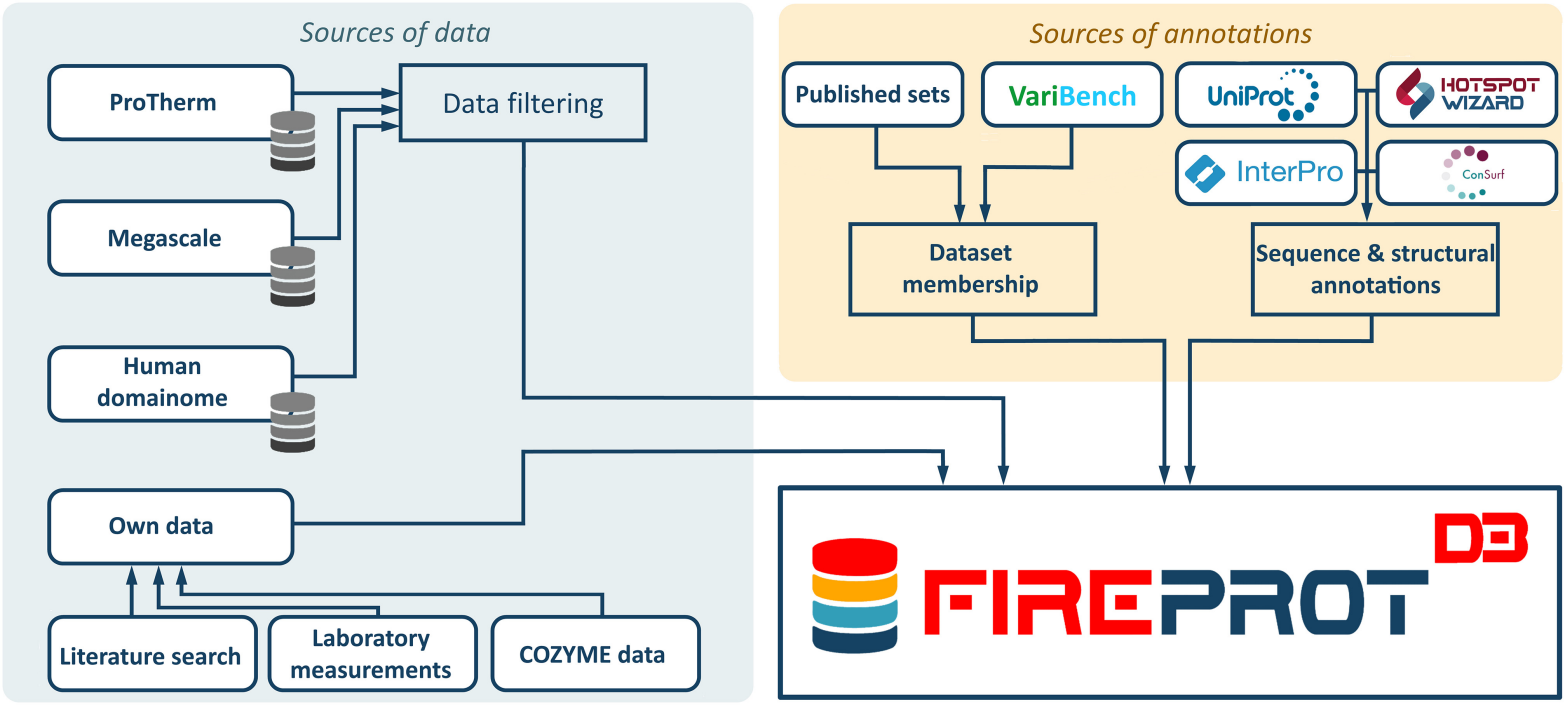
A schematic representation of data stored in the FireProtDB 2.0 database.

In addition to experimental measurements, the database has been enriched with annotations derived from several external tools and databases. The information on protein origin was retrieved from the UniProt database [44]. InterPro [45] was used to identify overlapping protein domains and families. ConSurf [46] was employed to generate multiple sequence alignments and estimate the evolutionary conservation of individual residues. Furthermore, structural features, such as pockets, tunnels, tunnel bottlenecks, and tunnel-lining residues, were annotated using Caver [47] and FPocket2 [48] implemented in the HotSpotWizard service [49]. Finally, a benchmarking repository of genetic variation datasets—VariBench [25]—designed to support the evaluation of computational prediction tools was utilized to annotate data with their presence in various protein stability datasets. This enables users to filter out the datasets used in the training of external computational models, which is particularly valuable for benchmarking. A full representation of data stored in the FireProtDB database is captured in Fig. 1.

### Database architecture

**Figure 2.**
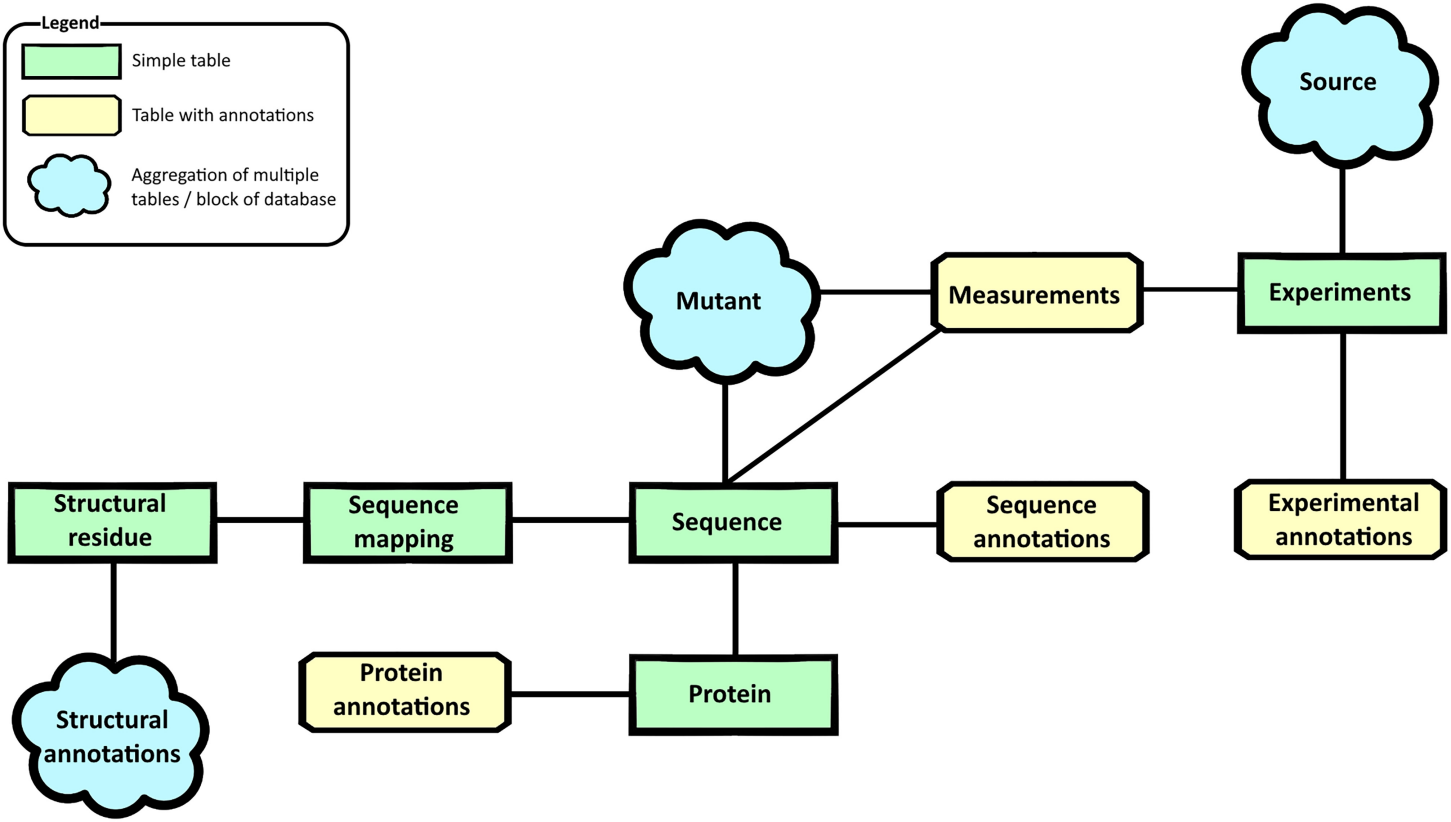
A schematic representation of the database architecture. Rectangular tables are the direct representation of the database entity with the chamfered rectangles being abstract key-value tables. Cloud shape represents a larger part of the database aggregated for simplicity. The Full Entity-relationship diagram can be found in the Supplementary material 1.

The FireProtDB database, implemented in PostgreSQL, comprises thirty tables, which can be organized into several logical blocks, as illustrated in Fig. 2. The database is sequence-centric, meaning that structural information is not required for data to be stored. Given that the new version is designed to accommodate both absolute and relative values, as well as a wide range of annotation types, the central reference point for each database entry is the native protein sequence. This sequence is preferably retrieved from the UniProt database [44], if available, or alternatively sourced from the original publication. In cases where only the mutant sequence is available, the native sequence is reconstructed based on the provided list of mutations. The *Mutant* entity encompasses a group of tables used to track the specific insertions, deletions, and substitutions applied to generate a given mutant sequence. It also captures hierarchical relationships between designs, particularly when a mutant is derived from another mutant rather than directly from the native sequence. The *Measurement* table stores both absolute values (e.g. ΔG, T_m_) linked to the native sequence, and relative values (e.g. ΔΔG, ΔT_m_) associated with the mutant. Since multiple measurements can originate from a single laboratory experiment, the *Experiment* is modeled as a distinct entity, allowing the inclusion of metadata such as pH, buffer composition, and concentration, denaturants, and other experimental conditions in the attached *Experimental annotations* table. The *Source* block contains tables describing the publications in which the data were first reported, including the list of authors. It also records the primary dataset and all additional datasets in which a given experimental entry has appeared.

Each *Sequence* in the database can be associated with multiple *Sequence annotations*, computed either directly from the sequence itself or derived from multiple sequence alignments of homologous proteins. Additionally, each sequence is linked to a single entry in the *Protein* entity, which stores information about the protein’s name and biological origin. Supplementary annotations—such as cross-references to external databases—are stored in a separate table, allowing for flexible integration of identifiers and metadata from various sources.

The final section of the database stores structural information, including protein tunnels, pockets, tunnel bottlenecks, and residue-specific B-factors. These data are linked not directly to the sequence, but to the corresponding tertiary structures representing the sequence. Multiple, slightly different structural models can be associated with a single sequence, each annotated independently. The central entity in this structural block is the *Structural residue*, which must be accurately mapped to the corresponding sequence residue, as structural and sequence residue numbering often differ. This mapping is performed using SIFTS [50]. The database also accommodates different biological assemblies, allowing the storage of multiple biological units derived from the same protein structure.

In addition to the main database, two materialized views have been created to enhance the efficiency of complex queries and data retrieval. The *Protein_view* aggregates information from across the entire database into a JSON format, where each entry represents a comprehensive JSON object containing all available data for a given protein. This design enables rapid loading and visualization of protein pages without the need to query multiple tables individually, significantly improving response times. The *Search_view* is specifically optimized to accelerate complex search queries. It consolidates arrays of searchable attributes associated with individual sequences with each column indexed using GIN indexing [51], thereby facilitating efficient filtering and retrieval. Each entry is also accommodated with a permanent unique identifier that is directly accessible in accordance with the FAIR principles [52, 53].

## Results

### Web interface

FireProtDB was primarily designed to enable fast and straightforward construction of protein stability datasets for training and validation, and benchmarking of novel computational tools. Nevertheless, its user interface is also well-suited for researchers outside the fields of machine learning and computational biology. A variety of data types are visually integrated along the sequence track, offering a comprehensive, in-depth view of each protein and its properties. The web server is organized into four main components: (i) a search engine for querying the database, (ii) search results displaying a summary of matching entries, (iii) a protein view presenting detailed information and visualizations for a selected protein, and (iv) a mutation view providing insights into individual variants and their associated experimental data. Alternatively, data can also be accessed via the API, documented at https://loschmidt.chemi.muni.cz/fireprotdb/api-docs/.

### Search engine

**Figure 3.**
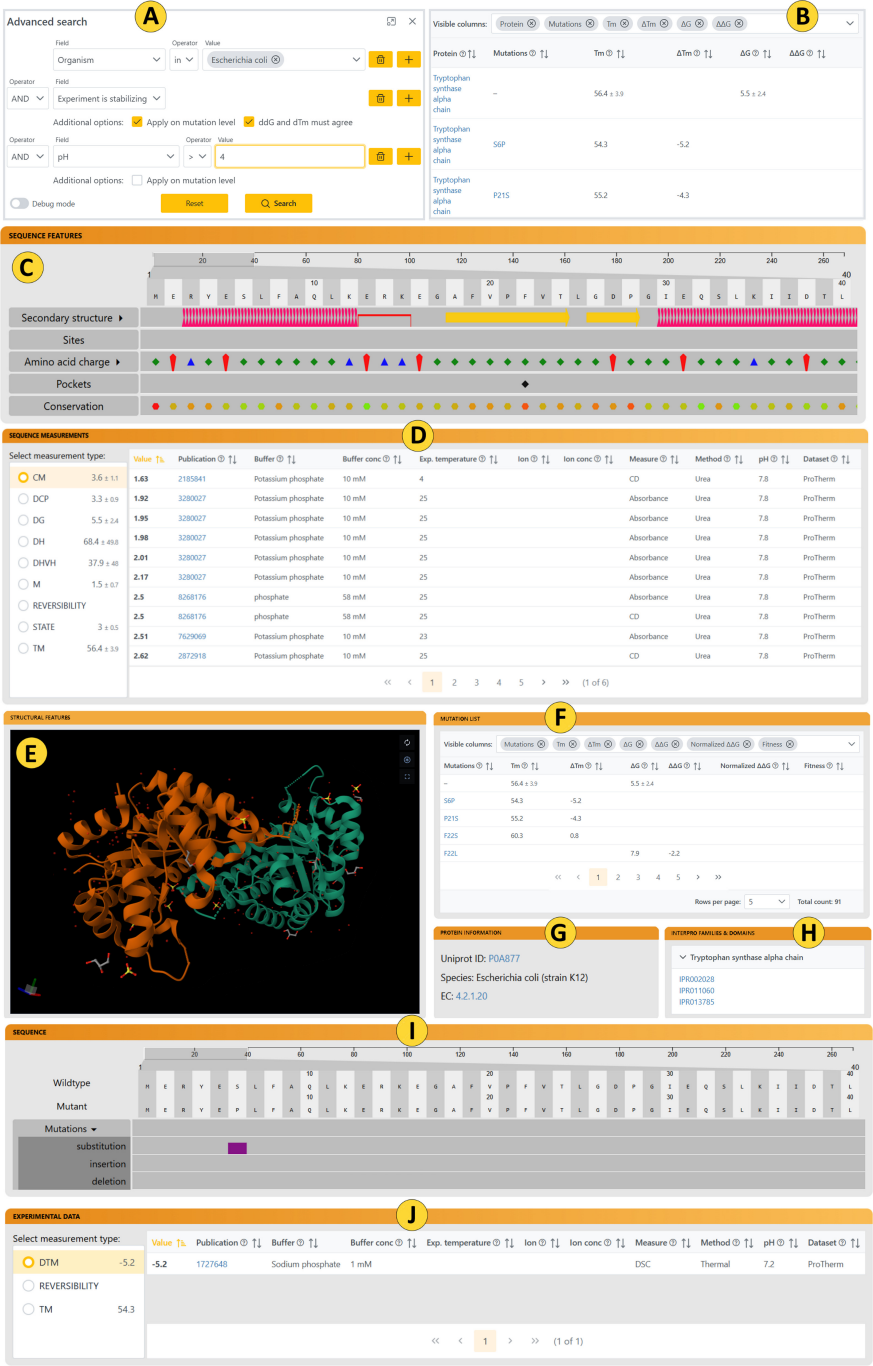
Graphical user interface of the FireProtDB 2.0 web server. The interface consists of several functional components: (**A**) Advanced search used for the construction of complex search queries. (**B**) A result table containing results fitting the search criteria. (**C**) Sequence track mapping various features directly on the sequence. (**D**) Table containing absolute values about the protein wild-type. (**E**) 3D structure visualization. (**F**) Mutant list showing all mutations attached to the protein. (**G**) Information about protein. (**H**) List of identified InterPro families. (**I**) Mapping of mutations on the protein sequence. (**J**) Distribution of ΔΔG across the protein sequence. (**K**) List of relative values connected to the selected mutant.

The search engine operates on two levels of interaction to accommodate both casual users and advanced researchers. At the most basic level, users can enter a search term into the main search bar. This initiates a standard full-text search across several data fields, including protein name, organism of origin, EC number, UniProt ID, PDB ID, InterPro ID, and publication. For more refined queries, users can click the “Advanced” button next to the search panel, enabling them to construct custom search conditions based on one or more criteria (Fig. 3A). The advanced search interface supports a total of 26 searchable fields, grouped into five distinct categories. Each field accepts one of the following input types: (i) a specific value for direct comparison, (ii) a value and a logical operator (e.g. equality, inclusion), or (iii) checkbox-based filters that define additional constraints.

Search terms can be combined using AND/OR logical operators, forming a tree-like query structure that is automatically translated into an optimized database query. This functionality makes the advanced search a powerful tool for extracting highly specific datasets. For example, users can retrieve all stabilizing mutations located in non-conserved regions of a given protein, or construct a benchmarking dataset consisting of mutations with experimentally validated ΔΔG values that were not used in training of the existing predictive models. The search engine, with several use cases, is further described in the Supplementary material 2.

### Search results

The results of a custom search are displayed in a results table (Fig. 3B), which by default includes several key measurements for each entry. Users can optionally expand the table to display up to ∼40 additional annotations and measurements, providing a more comprehensive view of the data. From the results table, users can directly access both the *Protein View* and *Mutation View* for any entry. Additionally, all selected data can be exported as a .csv file directly from the results page, facilitating downstream analysis and integration into external workflows.

### Protein view

The *Protein View* serves as the primary interface for exploring detailed information about a protein and its associated mutants. It is organized into several panels, each providing specific insights:

- Protein information: Displays key metadata including the protein’s origin, name, and relevant accession codes (Fig. 3G).
- Families and domains: Lists overlapping domains and families with direct links to the InterPro database for further exploration (Fig. 3H).
- Sequence features track: Offers an interactive visualization of various sequence- and structure-based features, mapped directly onto the protein sequence (Fig. 3C).
- Sequence measurements table: Presents all available experimental measurements related to the wild-type protein, specifically absolute values such as ΔG or T_m_ (Fig. 3D).
- 3D structure visualization: Provides a graphical representation of the protein’s tertiary structure, enabling spatial interpretation of functional or mutational data (Fig. 3E).
- ΔΔG visualization: Provides a graphical representation of the distribution of ΔΔG values across the protein sequence (Fig. 3J).
- Mutant table: Lists all known mutants of the protein along with their associated relative measurements (e.g. ΔΔG, ΔT_m_), allowing quick comparison and analysis (Fig. 3F).

### Mutant view

The Mutant View page can be accessed either directly from the search results table or via the mutant list on the corresponding *Protein View* page. It provides a more detailed analysis of a selected mutant, including all available experimental measurements related to the given variant (Fig. 3K). Additionally, the page features a sequence alignment between the wild-type and mutant sequences with all insertions, deletions, and substitutions clearly highlighted on the sequence track (Fig. 3I), allowing users to easily identify and interpret the introduced changes.

### Statistics

**Figure 4.**
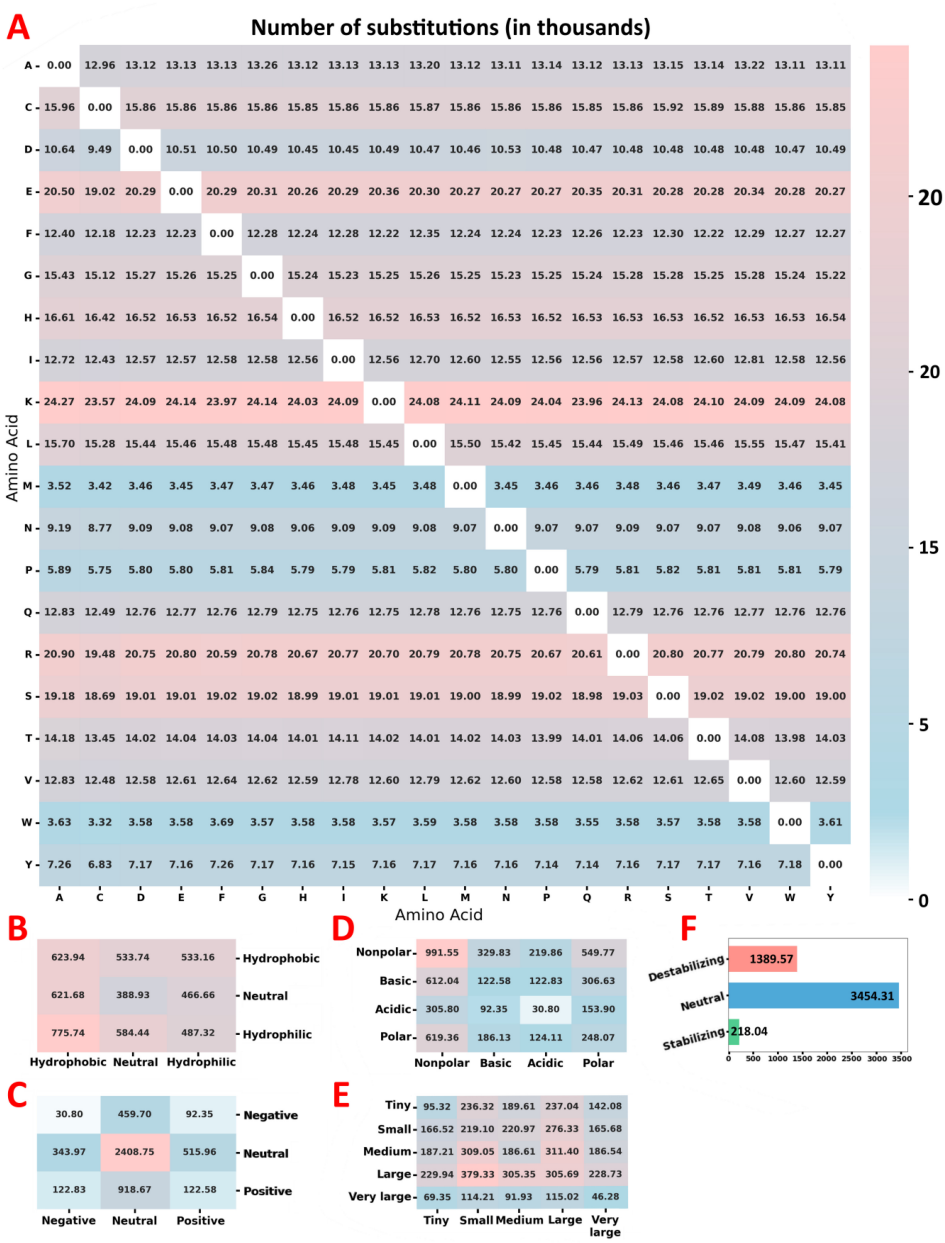
The statistics of the FireProtDB database, measured in thousands—the horizontal axis represents wild-type amino acids, and the vertical axis shows substitutions. (**A**) Amount of substitution data for each pair of amino acids; (**B**) substitutions based on the change in hydrophobicity; (**C**) substitutions considering charged amino acids; (**D**) substitutions causing a change in amino acid polarity; (**E**) substitutions based on the change in the size of the mutated amino acid; (**F**) a total number of stabilizing, destabilizing, and neutral mutations.

By integrating data from multiple sources, a total of 12 923 886 measurements, aggregated into 5 465 664 experiments, were compiled into the database. Of these, 42 912 measurements represent absolute values associated with wild-type protein sequences, while the remaining 12 880 974 characterize mutant proteins through relative measurements. The dataset encompasses 47 173 insertions, 50 383 deletions, 4 778 176 single-point substitutions, and 186 194 multiple-point substitutions. A detailed breakdown of substitution frequencies for each pair of amino acids is shown in Fig. 4A, along with aggregated data on substitutions categorized by amino acid hydrophobicity (**4B**), charge (**4C**), polarity (**4D**), and volume (**4E**)

Figure 4F illustrates the distribution of mutations based on their effect on protein stability, classified into stabilizing, neutral, or destabilizing groups. The categorization is based on four criteria—experimental ΔΔG [54] and ΔT_m_ values, as well as normalized ΔΔG and fitness scores as defined in Human Domainome dataset [32]—applied as follows:

- A mutation is considered stabilizing if it meets at least one of the following thresholds: experimental ΔΔG < −0.5 kcal/mol; normalized ΔΔG < −0.3; ΔT_m_ > 1°C; or fitness > 0.3.
- A mutation is considered destabilizing if it meets at least one of the following conditions: experimental ΔΔG > 0.5 kcal/mol; normalized ΔΔG > 0.3; ΔT_m_ < −1°C; or fitness < −0.3.
- A mutation is classified as neutral if it falls within at least one of the following ranges: experimental ΔΔG ∈ [−0.5, 0.5] kcal/mol; normalized ΔΔG ∈ [−0.3, 0.3]; ΔT_m_ ∈ [−1, 1]°C; or fitness ∈ [−0.3, 0.3].
- If more than one measurement (ΔΔG, ΔT_m_, normalized ΔΔG, and fitness) is available for a given mutation, all have to agree; otherwise, the mutation is considered inconclusive. This can be caused by differences in experimental conditions (pH, buffer, etc.), the imperfect correlation of ∼0.71 between ΔΔG and ΔT_m_ [55] (especially with mutations that are close to neutral), the average experimental measurement error of ∼0.48 kcal/mol [54], or by discrepancies in the source data. In such cases, the mutation will not be categorized as either stabilizing or destabilizing, unless explicitly specified by the user by limiting the search to only a selected type of measurement.

As expected, the dataset is dominated by neutral and destabilizing mutations, comprising 3 454 310 and 1 389 567 entries, respectively, compared to a smaller subset of 218 044 stabilizing mutations. Nevertheless, even this limited number of stabilizing variants represents a substantial dataset, sufficient for meaningful training, validation, and benchmarking of computational prediction tools.

## Conclusions

The availability of high-quality protein stability datasets is essential for the continued advancement of predictive computational tools. While the original FireProtDB 1.0 provided researchers with a substantial collection of manually curated data, it was limited by a rigid database architecture and restricted data scope. The new version of FireProtDB 2.0 addresses these limitations through a complete redesign of the database structure, adopting a more abstract and flexible model that supports the seamless integration of new data types. In addition to relative information on single-point mutations, the database now includes data on multiple-point mutations, insertions, deletions, and absolute measurements related to wild-type proteins. Importantly, the system is fully extensible, enabling the incorporation of new annotations and experimental measurements as they become available, ensuring that FireProtDB remains a scalable and valuable resource for the scientific community.

In total, the latest release of the FireProtDB 2.0 database comprises 12 923 886 measurements, aggregated into 5 465 664 experiments conducted on 2 762 unique proteins, thus increasing the number of experiments by two orders of magnitude compared to the original set of 15 987 mutations. This comprehensive dataset is the result of integrating data from previously published datasets, large-scale mutagenesis studies, literature mining, and in-house experimental work, including collaborations with partner laboratories through the COZYME initiative (https://cozyme.eu/). In addition to experimental data, the database includes a wide range of automatically generated annotations, sourced from external databases such as UniProt, InterPro, and VariBench, and computed using tools such as ConSurf and HotSpotWizard.

FireProtDB 2.0 provides an accessible and comprehensive platform for the construction of high-quality training and validation datasets for predictive tools, as well as for researchers seeking to analyze proteins of interest through an intuitive user interface. This dual functionality establishes FireProtDB 2.0 as an invaluable resource for both machine learning applications and fundamental biological research. Moreover, its flexible architecture ensures that FireProtDB 2.0 can evolve to accommodate future advances while maintaining fast and efficient complex query performance through optimized use of native database functions.

## Supplementary Material

gkaf1211_Supplemental_Files

## Data Availability

The sequences and measurements for the Human Domainome 1 dataset were obtained from https://zenodo.org/records/13629491 (Extended table 2 and 4), the MegaScale data are freely accessible at https://zenodo.org/records/7992926, and ProTherm data were downloaded from https://web.iitm.ac.in/bioinfo2/prothermdb/. Annotations of mutation presence in various training datasets were based on https://structure.bmc.lu.se/VariBench/data/effectspecific/stability.php, and protein annotations were automatically sourced from https://www.uniprot.org/ and https://www.ebi.ac.uk/interpro/ based on their UniProt access codes. Documentation for the FireProtDB API can be found at https://loschmidt.chemi.muni.cz/fireprotdb/api-docs/.
