# Supplementary material for: FireProtDB 2.0: large-scale manually curated database of the protein stability data
Source: Nucleic Acids Res. 2025 Nov 20;54(D1):D409–18. doi: 10.1093/nar/gkaf1211 (PMC12807726; doi:10.1093/nar/gkaf1211)

Search engine usage

The FireProt-DB database serves two primary functions: it is designed to facilitate the construction of robust datasets for training novel machine learning models and for benchmarking their performance against established methodologies. Concurrently, it offers an invaluable resource for biologists, enabling targeted searches for known experimental stability data pertaining to their specific protein of interest. This document provides a detailed description of several use cases, illustrating how the advanced search functionality can be precisely configured to achieve these distinct research objectives.

General setup

The Advanced Search interface facilitates the construction of complex queries by enabling the connection of **multiple subqueries** through the application of **Boolean operators (AND/OR).**

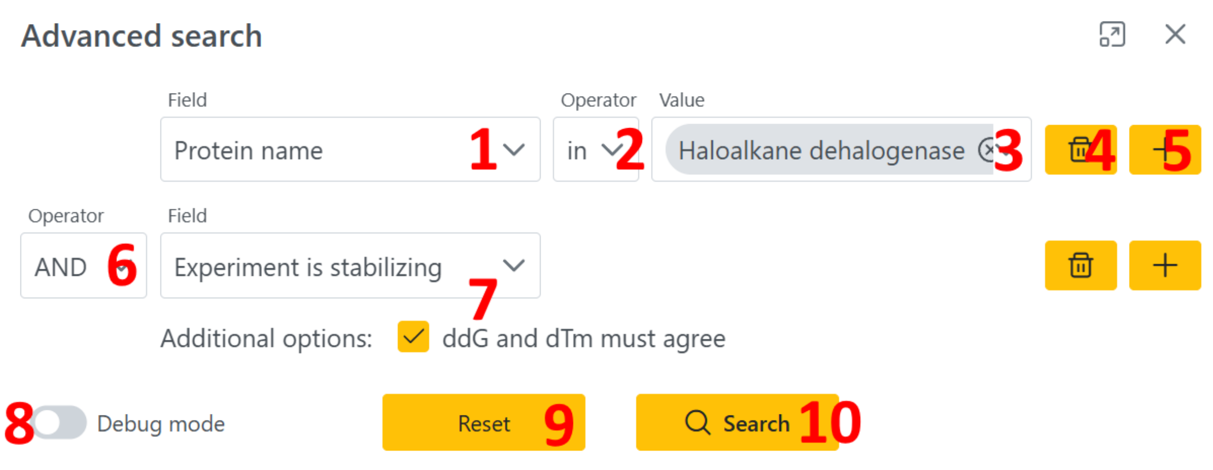

A single subquery is generally composed of three primary components: ***Field*** (1), ***Operator*** (2), and ***Value*** (3).

Subquery Components

- ***Field*** (1): Specifies the search parameter, such as protein name, UniProt ID, Dataset, or the type and effect of mutation.
- ***Operator*** (2): Determines the search logic and depends on the ***Field*** type. Common operators include inclusion (in/not in) or comparators (less than, greater than, equal).
- ***Value*** (3): Contains one or multiple searched values.

Note that not all fields require both an ***Operator*** and a ***Value***. For instance, fields searching for an exact match, such as UniProtKB ID, may omit the operator. Furthermore, unitary fields (e.g., 'experiment is stabilizing') are self-contained, as their value is intrinsically defined by the field itself, though ***extra settings*** (7) may still be available.

Query Management and Execution

The Advanced Search interface offers several tools for managing and executing the query:

- **Logic Connection**: New subqueries can be added via the ***plus icon*** (5) and must be connected to the existing query using a ***Boolean operator (AND/OR)***, following standard logical precedence (6).
- **Modification**: The ***bin icon*** (4) allows for the removal of a selected subquery.
- **Debugging:** The entire query structure can be reviewed by enabling ***debug mode*** (8) for visualization.
- **Execution**: The ***Search button*** (10) submits the finalized query to the database, while the ***reset button*** (9) clears the entire construction.

Use case 1

A researcher is interested in stabilizing the haloalkane dehalogenase protein without compromising its biological activity. Consequently, the search is configured to retrieve all known stabilizing mutations that are not located in close proximity to the protein's tunnel. The resulting query would be structured as follows:

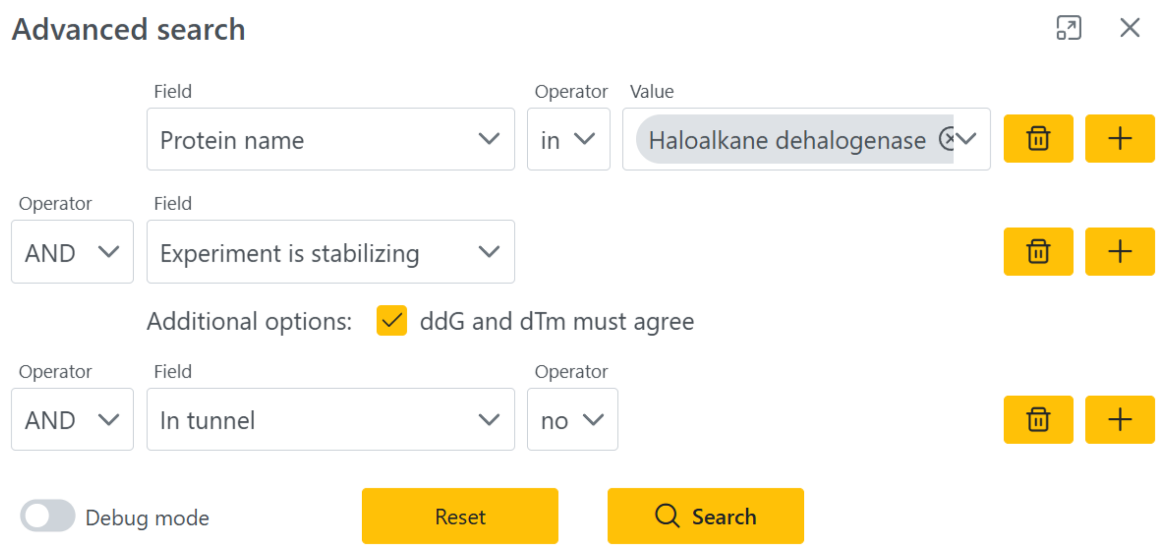

Use case 2

A researcher aims to benchmark a novel computational tool against existing methods while mitigating the risk of data overlap between the benchmark dataset and the training data used for the existing tools. Furthermore, the researcher is only interested in data points where protein stability is measured as ΔΔG, as this value is directly predicted by the selected computational tools.

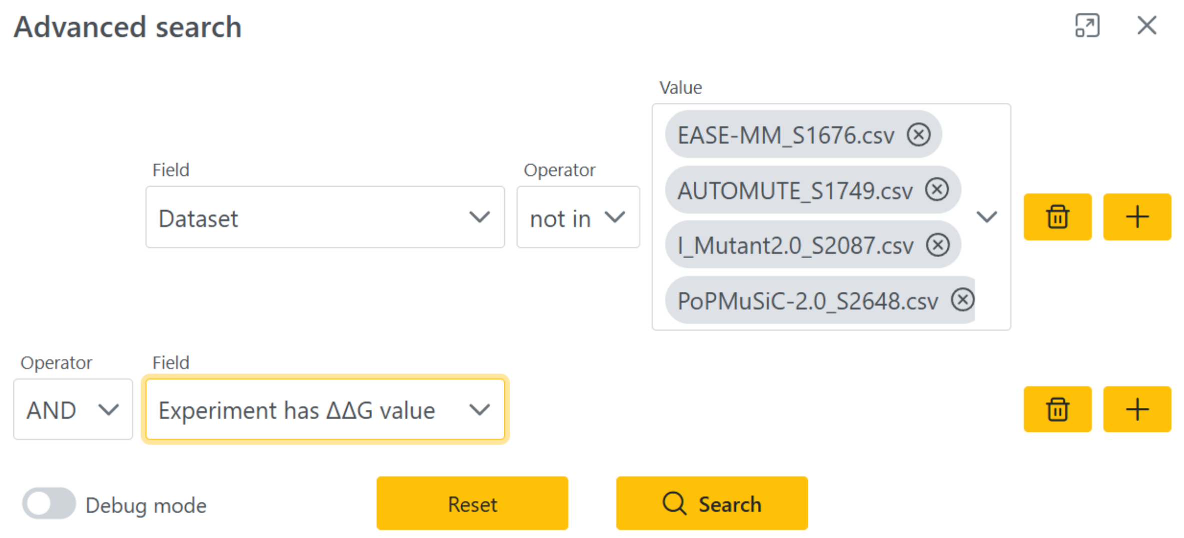

Alternatively, a simpler approach involves removing all data points originating from the ProTherm dataset, as ProTherm served as the primary training source for the majority of older computational tools. The resulting simplified query would be structured as follows:

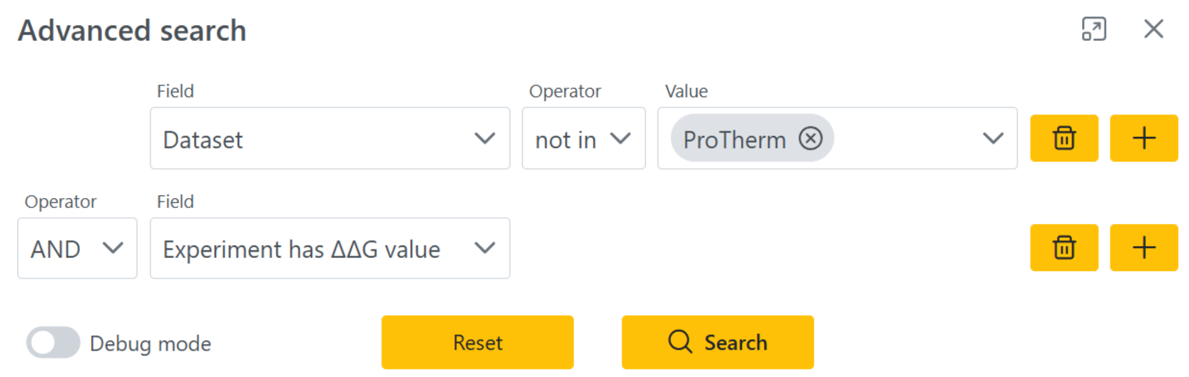

Use case 3

The goal is to find stabilizing mutations for Fibroblast Growth Factor 1 (FGF1) while restricting the results to data measured under specific physicochemical conditions. This objective requires three connected subqueries utilizing the AND operator to ensure all criteria are met simultaneously:

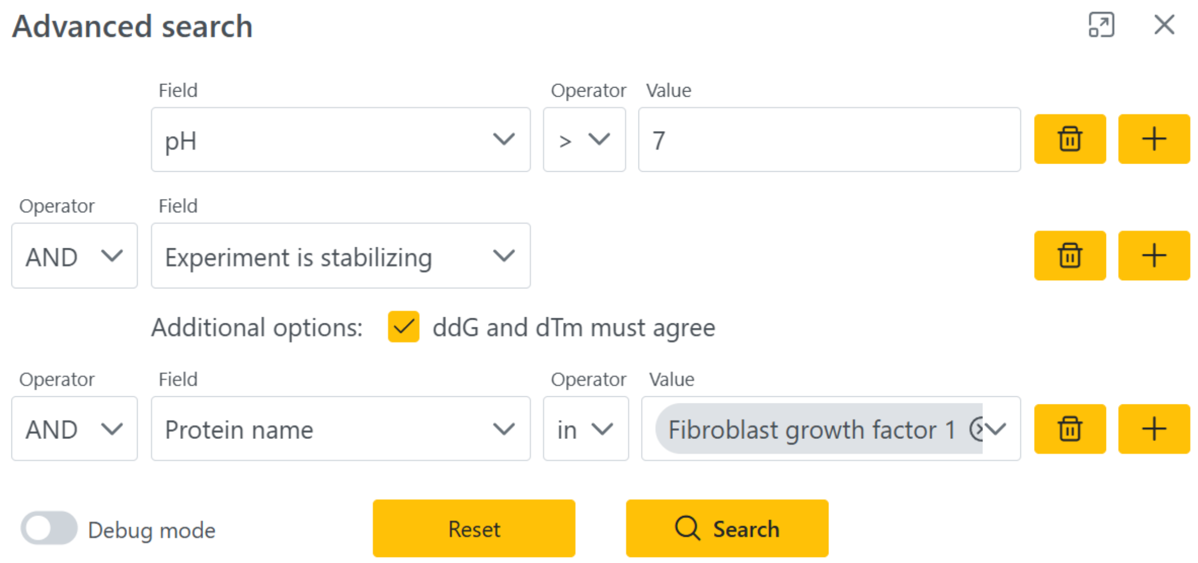

Use case 4

A researcher, aware that tryptophan (Trp) is frequently employed to fill protein cavities and thereby enhance stability, intends to query the database for all instances where tryptophan was used to occupy a pocket/cavity within the Thermonuclease protein. The query would be structured as follows:

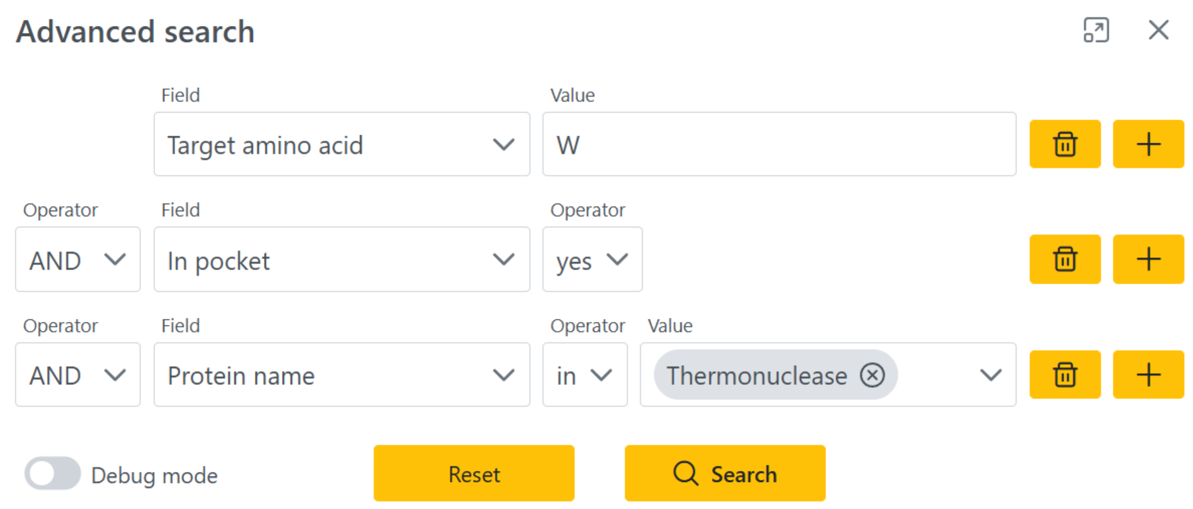

Use case 5

A researcher is interested in designing a predictive tool intended to enhance the stability of human proteins. To construct an appropriate training dataset for this purpose, the query would be structured as follows:

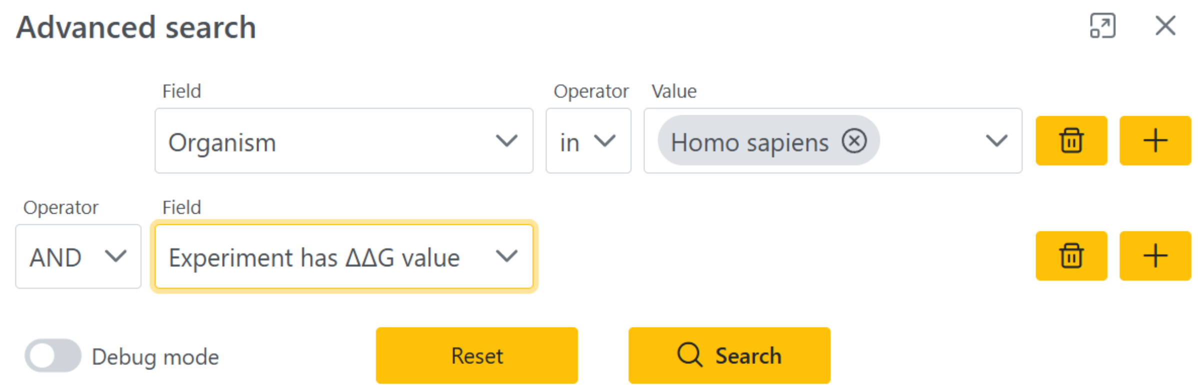

Use case 6

A researcher is interested in retrieving the list of mutations specifically used for the training of the PopMuSic predictor. To construct the query, the researcher needs to filter the database by the specific dataset(s) associated with PopMuSic's training:

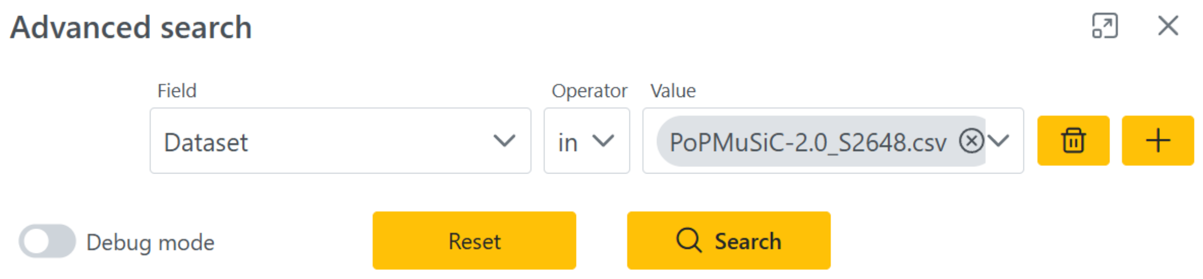

Use case 7

A researcher is interested in stabilizing a protein using multiple mutations. While stabilizing mutations are generally preferred, neutral mutations that have a negligible effect on stability (≈ΔΔG near 0), but offer other beneficial effects (such as improved activity or solubility), might also be useful to include in the design. To construct a comprehensive dataset that includes both positive-effect and neutral mutations, the query requires combining two distinct subqueries using the OR operator.

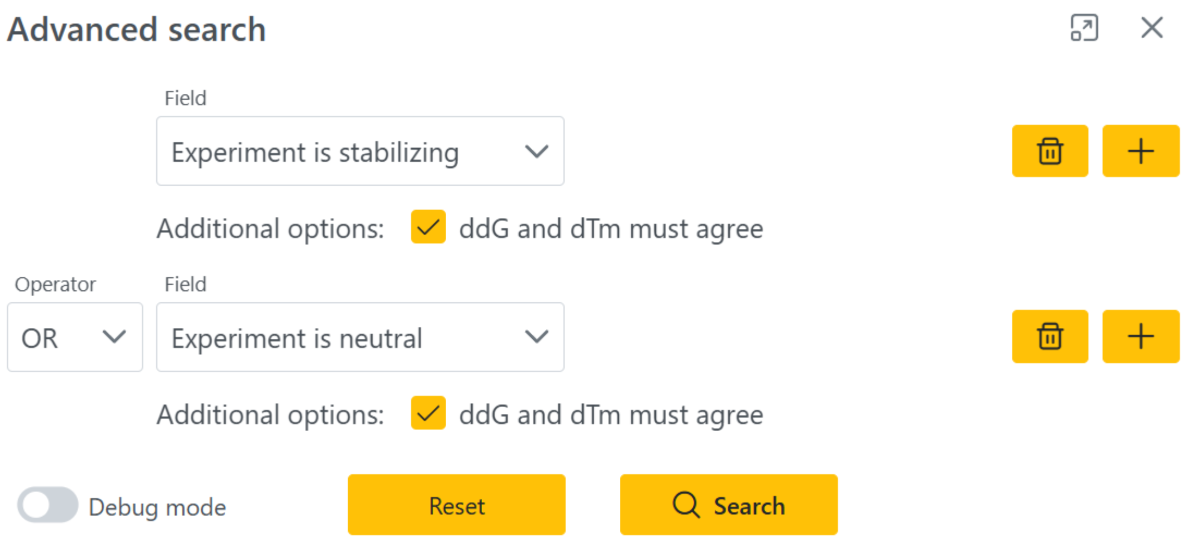

Supplement: gkaf1211_Supplemental_Files [file gkaf1211_supplemental_files.zip › Supplementary_material2.pdf]
