# Supplementary figures and images for: FireProtDB 2.0: large-scale manually curated database of the protein stability data
Source: Nucleic Acids Res. 2025 Nov 20;54(D1):D409–18. doi: 10.1093/nar/gkaf1211 (PMC12807726; doi:10.1093/nar/gkaf1211)

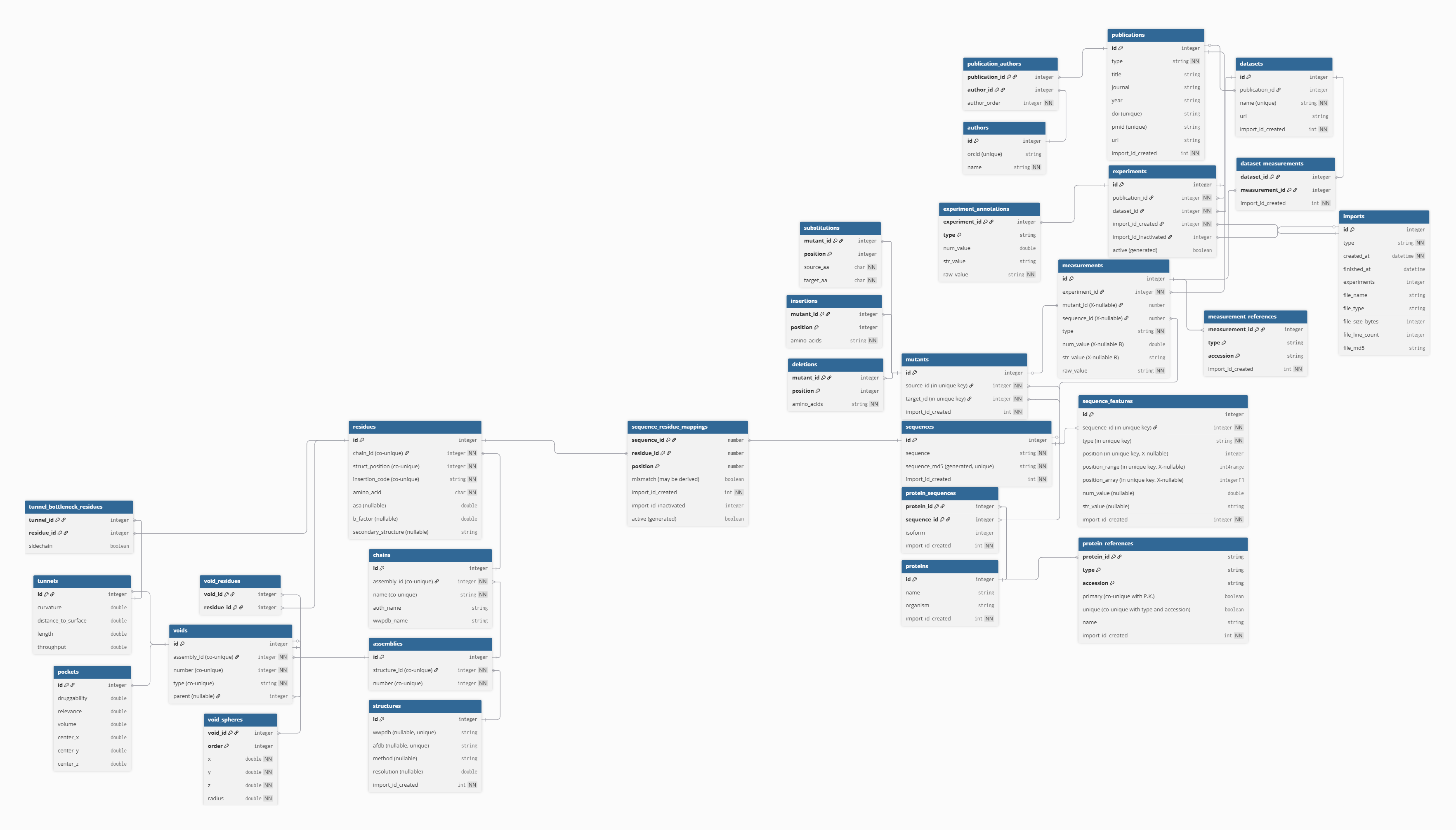

Supplement: gkaf1211_Supplemental_Files [file gkaf1211_supplemental_files.zip › Supplementary_material1.tif]
